# Apple Autotetraploids with Enhanced Resistance to Apple Scab (*Venturia inaequalis*) Due to Genome Duplication-Phenotypic and Genetic Evaluation

**DOI:** 10.3390/ijms22020527

**Published:** 2021-01-07

**Authors:** Małgorzata Podwyszyńska, Monika Markiewicz, Agata Broniarek-Niemiec, Bożena Matysiak, Agnieszka Marasek-Ciolakowska

**Affiliations:** 1 Department of Applied Biology, Research Institute of Horticulture, Konstytucji 3 Maja 1/3 Street, 96-100 Skierniewice, Poland; monika.markiewicz@inhort.pl (M.M.); bozena.matysiak@inhort.pl (B.M.); agnieszka.marasek@inhort.pl (A.M.-C.); 2 Department of Phytopathology, Research Institute of Horticulture, Konstytucji 3 Maja 1/3 Street, 96-100 Skierniewice, Poland; agata.broniarek@inhort.pl

**Keywords:** polyploidization, DNA methylation, disease-resistance related genes, *Rvi* genes, *Venturia inaequalis*, *Malus* × *domestica*

## Abstract

Among the fungal diseases of apple trees, serious yield losses are due to an apple scab caused by *Venturia inaequalis*. Protection against this disease is based mainly on chemical treatments, which are currently very limited. Therefore, it is extremely important to introduce cultivars with reduced susceptibility to this pathogen. One of the important sources of variability for breeding is the process of polyploidization. Newly obtained polyploids may acquire new features, including increased resistance to diseases. In our earlier studies, numerous tetraploids have been obtained for several apple cultivars with ‘Free Redstar’ tetraploids manifesting enhanced resistance to apple scab. In the present study, tetraploids of ‘Free Redstar’ were assessed in terms of phenotype and genotype with particular emphasis on the genetic background of their increased resistance to apple scab. Compared to diploid plants, tetraploids (own-rooted plants) were characterized with poor growth, especially during first growing season. They had considerably shorter shoots, fewer branches, smaller stem diameter, and reshaped leaves. In contrast to own-rooted plants, in M9-grafted three-year old trees, no significant differences between diplo- and tetraploids were observed, either in morphological or physiological parameters, with the exceptions of the increased leaf thickness and chlorophyll content recorded in tetraploids. Significant differences between sibling tetraploid clones were recorded, particularly in leaf shape and some physiological parameters. The amplified fragment length polymorphism (AFLP) analysis confirmed genetic polymorphism of tetraploid clones. Methylation-sensitive amplification polymorphism (MSAP) analysis showed that the level of DNA methylation was twice as high in young tetraploid plants as in a diploid donor tree, which may explain the weaker vigour of neotetraploids in the early period of their growth in the juvenile phase. Molecular analysis showed that ‘Free Redstar’ cultivar and their tetraploids bear six *Rvi* genes (*Rvi5*, *Rvi6*, *Rvi8*, *Rvi11*, *Rvi14* and *Rvi17*). Transcriptome analysis confirmed enhanced resistance to apple scab of ‘Free Redstar’ tetraploids since the expression levels of genes related to resistance were strongly enhanced in tetraploids compared to their diploid counterparts.

## 1. Introduction

The most important fruit species in the temperate climate zone is the apple tree (*Malus* × *domestica* Borkh.). Bacterial and fungal diseases are the greatest threats to apple cultivation in regions with a humid and cool climate. Among the fungal diseases of apple trees, serious yield losses are caused by apple scab, of which the causal agent is the ascomycete fungus *Venturia inaequalis* [1]. Protection against this disease is based mainly on chemical treatments and accounts for most of the total cost of protection against pests and diseases [2]. Due to Regulation (EC) No 1107/2009 of the European Parliament and Council [3], protection against apple scab is currently very limited. Therefore, it is extremely important to introduce cultivars with increased resistance to this disease into production [1,4].

Classical breeding methods focus on crossing cultivars for pyramid genes associated with different resistance mechanisms. For resistance to apple scab, wild species of the genus *Malus* are considered potential sources of resistance in breeding. Several qualitative and quantitative trait loci (QTL) have been identified, which are responsible for polygenic-conditioned traits, conferring some degree of resistance [1,5]. Genetic resistance to *V. inaequalis* relies on gene-for-gene interactions [6], in which infection development is influenced by the interaction between the plant resistance gene (*R*) and a gene from the pathogen, avirulence gene (*Avr*). In such interactions, the product of a particular *Avr* is recognized by the plant-specific suitable *R* gene. In cases where the plant bears the susceptibility allele or if the pathogen possesses the specific virulence allele, infection takes place. A set of differential hosts has been established and modified, including one for each of the apple scab resistance genes described in the new nomenclature system [1]. The most well-known *R* gene to this disease is *Rvi6* (*Vf*), derived from *Malus floribunda* 821 [7]. Currently, as recently reviewed by Khajuria et al. [5], a total of 20 *R* genes are known to be related with varying degrees of resistance to scab, including: *Rvi15* (*Vr*), *Rvi2* (*Vh2*), and *Rvi4* (*Vh4*) from *M. pumila* R12740-7a, *Rvi11* (*Vbj*) hosted by genotype A722–7 (*M. baccata jackii* × ‘Starking’), *Rvi10* (*Va*) from cultivar ‘Antonovka’ PI172623, *Rvi1* (*Vg*) from ‘Golden Delicious’, and *Rvi5* (*Vm*) from *M. micromalus*. Other hosts bearing *R* genes are ‘Dülmener Rosenapfel’ (*Rvi 14*), GMAL2473 (*Rvi15*), MIS op 93.051 G07–098 (*Rvi 16*), and ‘Antonovka’ APF22 (*Rvi 17*). This new system better reflects the emerging complexities associated with different combinations of genes, both in the host and pathogen. Resistance achieved by the presence of genes, *Rvi1* (*Vg*), *Rvi6* (*Vf*), *Rvi5* (*Vm*), *Rvi10* (*Va*), *Rvi11* (*Vbj*), *Rvi12* (*Vb*), and *Rvi15* (*Vr*), is racially specific and broken by mutated races of the pathogen in conditions of commercial cultivation [1]. Therefore, in many research centres worldwide, extensive studies are conducted to obtain new apple genotypes with enhanced resistance to this dangerous disease [4]. Achieving durable resistance through traditional breeding is a long-term process but can be accelerated by adding resistance into existing high-quality cultivars by marker-assisted fast breeding combined with other methods, including polyploidization.

One of the important sources of variability for breeding is the process of polyploidization [8,9,10]. Whole genome duplication is one of the most crucial pathways in introducing speciation and broadening biodiversity. Due to the occurrence of more copies of alleles at a locus, polyploids may have unique gene regulations, such as silencing or upregulation of the expression level to increase abiotic stress tolerance and biotic stress resistance [11]. Polyploidization causes not only a multiplication of the number of the same genes, including genes related to resistance, it is also a source of other types of genetic alteration, such as chromosomal mutations (deletions, translocations, and inversions), point mutations, and changes in DNA methylation patterns [9,11,12,13].

Polyploids are usually characterized by better phenotypic features, for example, vigorous growth, larger flowers and fruits, compact habit, and increased adaptability to biotic or abiotic stress factors [12,14,15]. Additionally, apple tetraploids have been reported to be characterized by higher fruit quality and increased resistance to biotic and abiotic stressors [16,17,18,19,20,21,22,23,24].

As a result of our experiments carried out at the Research Institute of Horticulture (RIH) (Skierniewice, Poland) in the years 2014–2020, numerous tetraploids have been obtained for six apple cultivars: ‘Free Redstar’, ‘Gala Must’, ‘Pinova’, Co-op 32, ‘Redchief’, and ‘Sander’ [25]. To obtain actively growing plants for further research, microcuttings of tetraploids were rooted according to an optimized procedure [26]. Preliminary phenotypic observation revealed that single tetraploid clones of ‘Gala Must’, ‘Pinova’, ‘Redchief’, and all of the tetraploids of ‘Free Redstar’ had increased resistance to apple scab and/or fire blight, as estimated in a greenhouse test and in vitro bioassay, respectively [23,24].

Plants have developed complex, multicomponent mechanisms that mitigate the attack of pathogens. The first line of defense is passive resistance associated with physical barriers and constitutive chemical resistance [27,28]. The second line of plant defense systems is triggered by pathogen-associated elicitors and consists of three steps: pathogen recognition, signal transduction, and activation of defensive response involving many pathways. 

To evaluate the genetic background of phenotypic alterations between apple ‘Free Redstar’ neotetraploids and their diploid counterparts, we determined the presence of various *Rvi* genes using cDNA-AFLP (amplified fragment length polymorphism) analysis, as well as examined the differential expression of *Rvi6* gene and other resistance-related genes, such as *PR1* (pathogenesis-related protein 1), *PR2* (β-1,3-glucanase), *WRKY29* (WRKY transcription factor 29), *CDPK* (calcium-dependent protein kinase), and *MPK4* (a mitogen-activated protein kinases). Previously, we successfully used cDNA-AFLP analysis to identify the resistance-related genes in *Malus* × *domestica* and *Prunus domestica* under infection by *Erwinia amylovora* and the *Plum pox virus*, respectively [29,30].

*PR1* with antioomycete and antifungal properties and *PR2* belonging to pathogenesis-related (PR) proteins are the key components of plant innate immune systems, especially systemic acquired resistance (SAR), and are widely used as the diagnostic molecular markers of defense signalling pathways [31]. *WRKY29*, *CDPK*, and *MPK4* genes are involved in plant resistant to fungal diseases. The *WRKY29* encoding transcription factor is involved in the expression of defense genes in the resistance response of plants. According to Sarowar et al. [32], an upregulated expression of *WRKY29* in plants enhances disease resistance. CDPKs are a kind of regulatory protein transmitting calcium ions (Ca^2+^), a secondary messenger in plant signalling systems [33]. They are involved in reactions that include the synthesis of reactive oxygen species (ROS), alteration of gene expression, changes in phytohormone synthesis, and signalling and cell death [34]. Mitogen-activated protein kinase (MAPK) pathways regulate signal transduction from different cellular compartments and from the extracellular environment to the nucleus in all eukaryotes. One of these proteins is MAP kinase 4 (MPK4), which was shown to be a regulator of systemic-acquired resistance and is activated by pathogen-associated molecular patterns (PAMPs) [35].

The aim of our research was to perform a general survey of the phenotypic and genetic changes, with particular emphasis on the genetic background of increased resistance to apple scab, in newly formed apple tetraploids of ‘Free Redstar’ in relation to their diploid counterpart. To determine genome alterations following chromosome doubling, AFLP analysis was performed. Possible changes in the DNA methylation rate were analysed using the methylation-sensitive amplification polymorphism (MSAP) method. We also attempted to confirm, in successive greenhouse tests, high resistance to apple scab of the ‘Free Redstar’ apple tetraploids obtained and to recognize the molecular mechanisms of increased resistance to this disease by determining *Rvi* genes, as well as the expression levels of various genes related to different resistance responses under infection with *V. inaequalis*.

## 2. Results

### 2.1. Morphological Observations

**Figure 1:**
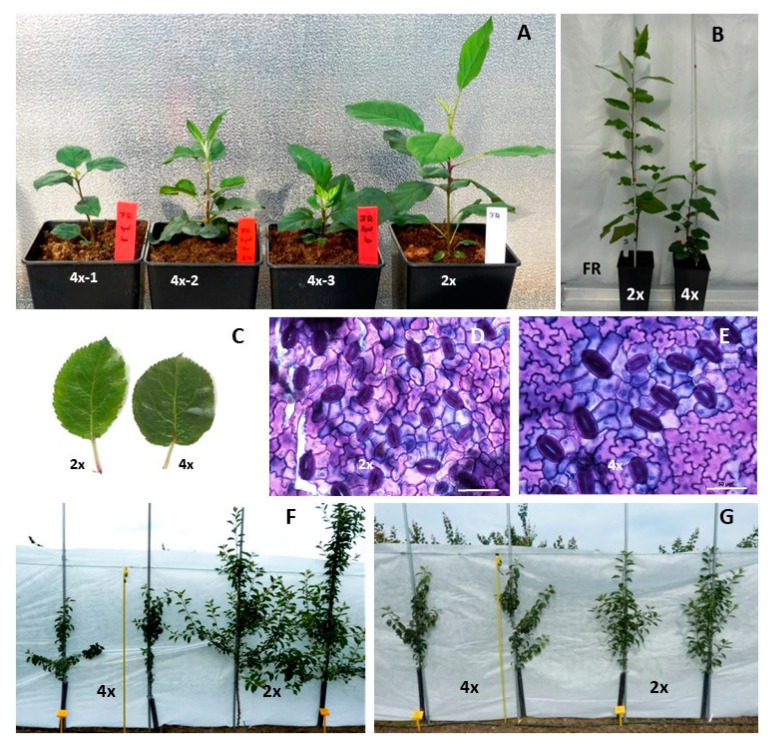
Phenotype of apple diploid and tetraploid plants of ‘Free Redstar’ cultivar: (**A**) three-month old and (**B**) six-month old own-rooted plants, (**C**) leaves and (**D**,**E**) stomata, (**F**) own-rooted and (**G**) M9-grafted three-year old plants growing in experimental orchard of The Research Institute of Horticulture (Skierniewice, Poland), bars represent 50 µm.

**Figure 2:**
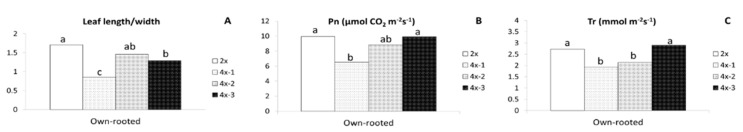
Morphological and physiological parameters of the ‘Free Redstar’ apple diploid and its tetraploid clones evaluated in six-month own-rooted plants; only parameters for significant differences between individual tetraploids were observed are presented: (**A**) leaf/length ratio, (**B**) net photosynthesis-Pn, (**C**) transpiration rate-Tr; means for each trait marked with the same letter do not differ significantly at *p* = 0.05; Duncan’s test.

Preliminary observation of three-month old plants revealed large differences between diploids and tetraploids, especially in shoot length and leaf shape (Figure 1A). Later evaluation of six-month old own-rooted plants (performed in 2018) showed that the highest differences in morphology between diploid and tetraploids were observed for plant height (Table 1, Figure 1B). Compared to diploids, tetraploids were on average three times shorter (14.5 cm), had two times fewer nodes (16.7), and the stem diameter was significantly smaller. Tetraploid leaves had a similar area and width to their diploid counterparts, however, the leaves differed considerably in shape, and they were more round as evidenced by a length to width ratio that was, on average, significantly lower in tetraploids (1.3) than in diploids (1.71) (Table 1, Figure 1C). Tetraploids had larger stomata (33.6%), and their leaves contained more chlorophyll (20%) than those of diploids (Table 1, Figure 1C–E). Although individual tetraploid clones were not significantly different from each other in shoot length, stem diameter, and leaf area, significant variation was found in leaf shape, where in leaves of tetraploid 4x-1 were more round compared to the remaining ones. The leaf length/width ratios were 0.86, 1.46, 1.29, and 1.71 for tetraploids 4x-1, 4x-2, 4x-3 and diploid, respectively (Figure 2A). Significant variation among young tetraploid clones was also recorded for physiological parameters (see Section 2.2).

**Figure 3:**
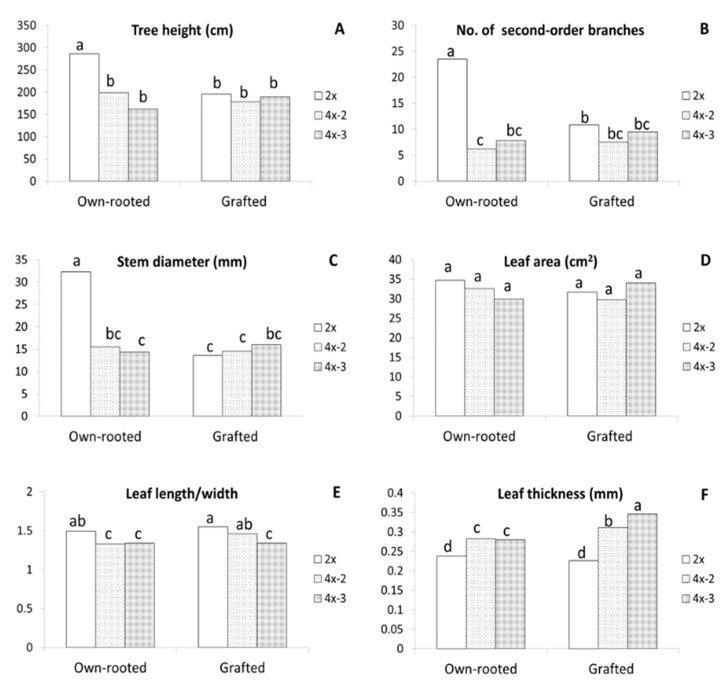
Morphological parameters of the ‘Free Redstar’ apple diploid and its tetraploid clones evaluated for the own-rooted and M9-grafted three-year old trees growing in an experimental orchard of the Research Institute of Horticulture (Skierniewice, Poland) (RIH): (**A**) stem height,(**B**) number of second number branches, (**C**) stem diameter, (**D**) leaf area, (**E**) leaf length/wide ratio, (**F**) leaf thickness; means for each trait marked with the same letter do not differ significantly at *p* = 0.05; Duncan’s test.

Evaluation of the three-year old plants growing in orchards made in 2020 showed that the largest differences between diploids and tetraploids were observed for own-rooted plants (Figure 1F). Compared to diploid plants, own-rooted tetraploid clones were significantly shorter, by approximately 30–40%, had three times less second-order branches (from 6 to 8), and the shoot diameters were more than two times smaller (Figure 3A–C). In contrast, no differences were observed between diploid and tetraploid M9-grafted plants in tree height, stem diameter, second-order branches, and leaf area (Figure 1G and Figure 3A–D). Although no differences were recorded in leaf area, irrespective of ploidy level and tree type, the leaves of tetraploids were more round, as evidenced by the significantly lower length/width ratio observed in them compared to that observed in diploid plants (Figure 3E). Tetraploid leaves were considerably thicker than those of diploid plants, moreover, the thickest leaves were recorded for grafted tetraploids (Figure 3F). Interestingly, in grafted plants, leaf shape of one tetraploid clone 4x-3 was more round compared to leaves of 4x-2. This is evidenced by significantly lower leaf length/width ratio of 4x-3 (1.34) than those of 4x-2 (1.46) and 2x (1.55).

### 2.2. Physiological Parameters 

Measurements performed in 2018 for six-month old own-rooted plants showed that chlorophyll content index (CCI) was on average higher in tetraploids by 20% compared to diploids (Table 1). Other physiological parameters, such as net photosynthesis rate (Pn), transpiration rate (Tr), and water-use efficiency (WUE), were on average comparable in diploid and tetraploid plants. Whereas quantum efficiency of open photosystem II centres (Fv/Fm) was significantly lower and photosynthetic quantum yield (Φ_PSII_) was much higher in tetraploids compared to diploids. However, individual tetraploid clones differed considerably between each other in Pn and Tr (Figure 2B,C). 

**Figure 4:**
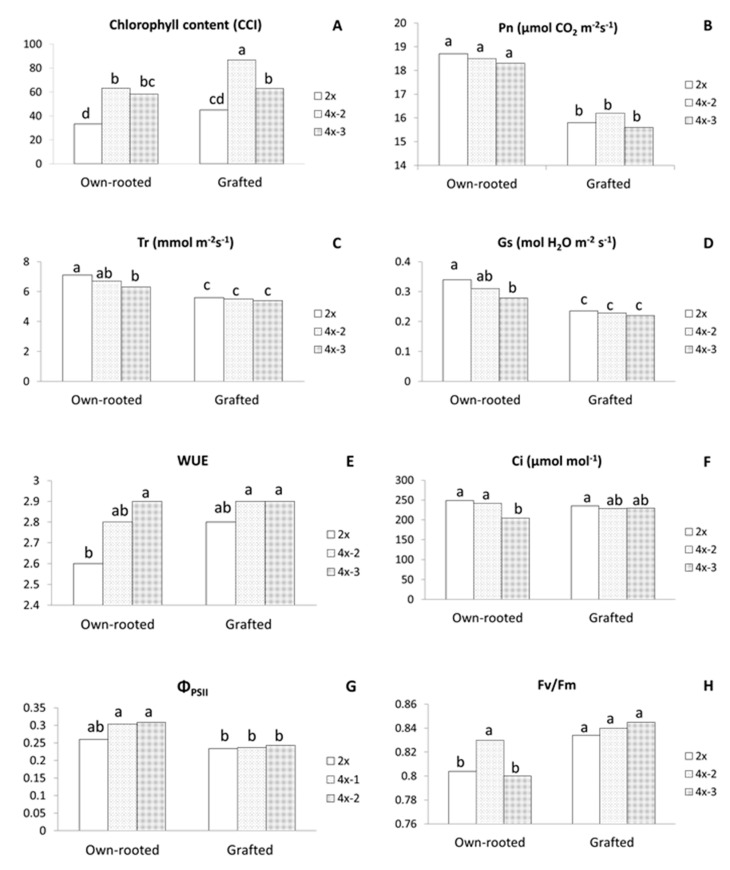
Physiological parameters of the ‘Free Redstar’ apple diploid and its tetraploid clones evaluated for the own-rooted and M9-grafted three-year old trees growing in the Research Institute of Horticulture (Skierniewice, Poland) (RIH) experimental orchard; (**A**) chlorophyll content index (CCI), (**B**) net photosynthetic rate (Pn), (**C**) transpiration rate (Tr), (**D**) stomatal conductance (Gs), (**E**) water-use efficiency (WUE), (**F**) and internal CO_2_ concentration (Ci), (**G**) photosynthetic quantum yield (Φ_PSII_), and (**H**) quantum efficiency of open photosystem II centres (Fv/Fm); means for each trait marked with the same letter do not differ significantly at *p* = 0.05; Duncan’s test.

In three-year old plants growing in an orchard in 2020, chlorophyll content was significantly higher in all tetraploids, either in own-rooted or grafted plants compared to diploids (Figure 4A). The highest CCI of 86.7 was recorded for grafted 4x-2 and the lowest of 30.0 CCI for own-rooted diploids. 

Among the gas exchange parameters, Pn, Tr, and stomatal conductance (Gs) were significantly lower for grafted plants irrespective of ploidy level (Figure 4B–D). Furthermore, in grafted plants, tetraploids did not differ in any parameter of gas exchange with WUE (2.8–2.9) being comparable to own-rooted tetraploids and significantly higher than WUE of own-rooted diploids (2.6) (Figure 4E). In own-rooted plants, tetraploid 4x-3 was characterized with significantly lower Tr, Gs, and internal CO_2_ concentration (Ci) compared to own-rooted diploids, but WUE of this tetraploid was much higher than that of the own-rooted diploid (Figure 4C–F). Analysis of chlorophyll fluorescence revealed that Φ_PSII_ values were similar for the grafted diploid and tetraploid plants, and at the same time, were all significantly lower compared to own-rooted tetraploids (Figure 4G). In contrast, Fv/Fm of diploid and tetraploid grafted plants were similar to each other and considerably higher than own-rooted diploids and 4x-3 (Figure 4H).

### 2.3. Susceptibility to Apple Scab

None of the ‘Free Redstar’ tetraploid plants showed symptoms of leaf infection, while the leaf lesion for the diploid counterpart was rated from 1.5 in 2017 to 4.3 in 2019. The reference ‘Idared’ rated from 2.0 to 4.0, which did not differ significantly from diploid progenitor ‘Free Redstar’, except in 2017, when leaves of ‘Idared’ were infected to a much greater extent (Table 2).

### 2.4. Analysis of Genetic and Epigenetic Changes in Tetraploids Compared to the Diploid Genotype

**Figure 5:**
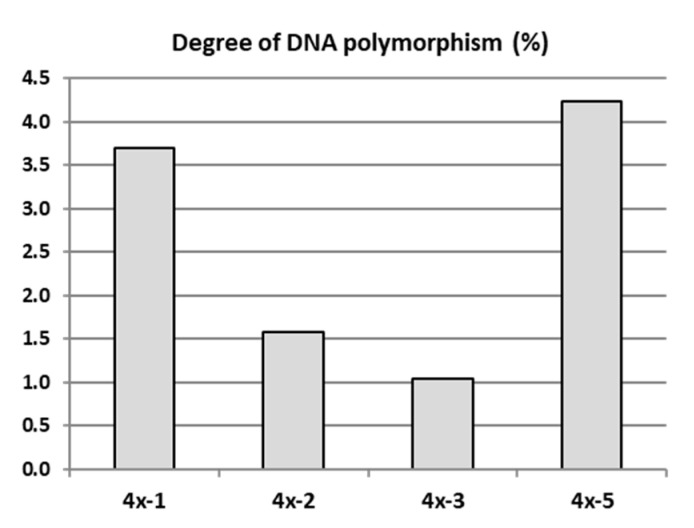
DNA polymorphism of apple tetraploid clones derived from the ‘Free Redstar’ cultivar determined with amplified fragment length polymorphism (AFLP) markers.

The genetic evaluation of four ‘Free Redstar’ autotetraploid clones differing in leaf shape and physiological parameters (4x-1, 4x-2, 4x-3, 4x-5) in relation to their diploid counterpart (2x) were carried out using the AFLP method in 2018. Randomly selected own-rooted six-month old plants of each tetraploid clone growing in a greenhouse and donor diploid plants growing in the experimental orchard were analysed. From 10 tested AFLP primer pairs used for the initial screening (unpublished data), 5 primer pairs that produced reproducible and scorable bands were used to analyse genetic changes in tetraploid plants compared to their diploid counterparts (Table S1). The number of bands generated by these primer pairs varied from 24 to a maximum of 46 amplification products, ranging in size from 120 to 700 bp (Table S2). The primer pair *Pst*-AT/*Mse*-AT, amplified the maximum number of AFLP markers, and the primer pair *Pst*-AA/*Mse*-AC, amplified the lowest number of AFLP markers. The mean degree of genetic differentiation of the tested tetraploids with AFLP markers was 2.43% in relation to the diploid counterparts. The higher variation of 3.70% and 4.23% was observed for tetraploid clones 4x-1 and 4x-5, respectively (Figure 5). These two tetraploid clones were less vigorous, and most of plants died. Therefore, these clones were not evaluated further.

**Figure 6:**
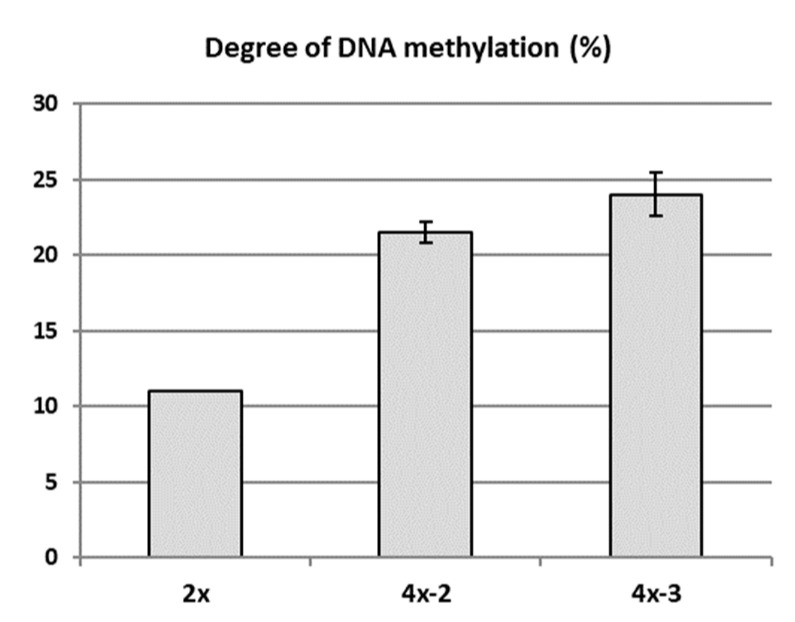
DNA methylation rate of apple diploid donor plant of ‘Free Redstar’ and its tetraploid clones 4x-2 and 4x-3. Error bars represent standard deviations.

Possible epigenetic changes occurring in the genome of two tetraploids (4x-2 and 4x-3) in relation to diploid counterparts (2x) were analysed using the MSAP in 2018. Randomly selected in vitro plantlets of each tetraploid clone, as well as the own-rooted six-month old plants growing in a greenhouse and the diploid donor plants growing in the experimental orchard were analysed. MSAP analysis revealed a higher DNA methylation rate detected in both tetraploid clones compared to their diploid counterparts (Figure 6). Average DNA methylation rates in the diploid and tetraploid clones were 11.0% and 22.75%, respectively. Clone 4x-3 was characterized with the highest DNA methylation rate (24%).

**Figure 7:**
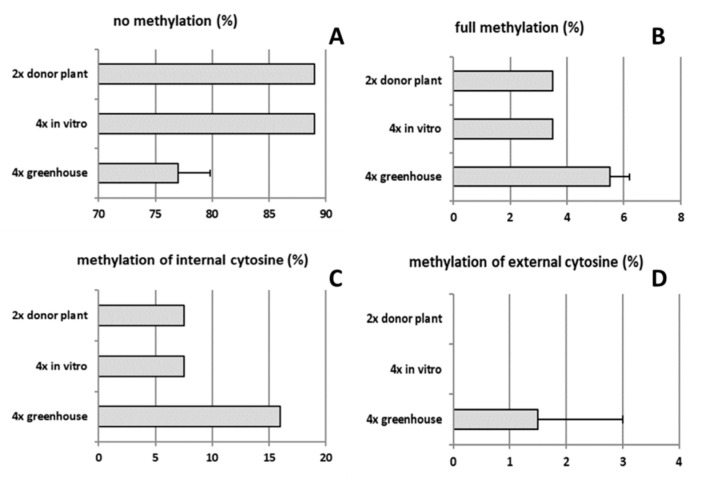
Type of DNA methylation in tetraploid apple cultivar ‘Free Redstar’: (**A**) no methylation, (**B**) full methylation, (**C**) methylation of internal cytosine, and (**D**) methylation of external cytosine. Error bars represent standard deviations.

The type of methylation was also assessed, both in diploid and tetraploid plants in various development stages (Figure 7A–D). DNA methylation rates of both tetraploid clones 4x-2 and 4x-3 were on average approx. two-fold higher compared to diploids. In tetraploid plants of in vitro culture and diploid donor plant, DNA methylation rates were twice lower (11%) compared to 6-month old tetraploids (grown in a greenhouse) (23%). In these tetraploid plants, the methylation of internal cytosine and full methylation were increased by 16.0 and 5.5%, respectively, compared to in vitro plant material and diploid donor plants.

### 2.5. Analysis of Rvi Genes

Results of *Rvi* genes’ analysis of ‘Free Redstar’ tetraploid clones in comparison to their diploid counterparts and sensitive to apple scab reference ‘Idared’ are presented in Table 3. The lengths of products ranging in length from 100 to 1100 bp, expected and unexpected, generated by primers corresponding to *Rvi* genes are presented in Table S3. Regions conjugated with apple scab resistance are in Table S3 marked as ‘R’. The polymerase chain reaction (PCR) primers: Vm specific to *Rvi5*; ACS7 and ACS9 specific to *Rvi6*; OBP12 and OPL19 specific to *Rvi8*; K08, T06 and Z13 specific to *Rvi11*; AD13 specific to *Rvi15*; and H-Vf1 specific to *Rvi17* generated the amplification products of expected lengths in diploid and all tetraploid clones. This indicated the existence of the aforementioned *Rvi* genes in analysed genotypes. There was, however, one exception in the diploids, for which one of the specific *Rvi6* products were not obtained with the ACS7 primer.

### 2.6. Comparative Transcriptome Analysis of Diploid and Tetraploid Apple Plants Inoculated with V. inaequalis (cDNA-AFLP (Amplified Fragment Length Polymorphism) Analysis)

**Figure 8:**
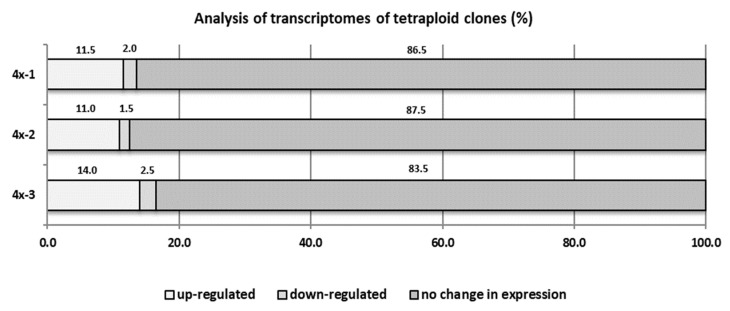
cDNA-AFLP analysis for three ‘Free Redstar’ apple tetraploid clones 4x-1, 4x-2, and 4x-3 compared to their diploid (2x) counterpart during *V. inaequalis* infection.

Inoculation with *V. inaequalis* caused significant changes in the transcriptome of the tetraploid plants. Our analysis showed that in tetraploid clones, on average 12.2% cDNA-AFLP products were overexpressed and 2% were silenced, compared to diploid plants (Figure 8). In our preliminary study, 30 upregulated sequence tags (dESTs) (cDNA-AFLP products) were identified in tetraploid clones of the ‘Free Redstar’ cultivar as compared with the diploid counterpart during *V. inaequalis* infection (Table 4). Basic Local Alignment Search Tool (BLAST) analysis revealed that 83.3% of these dESTs have homology to known genes encoding proteins. Three of them (M17, M21, and M25) encoded unknown proteins. The remaining dESTs encoding proteins were involved in gene expression regulation (HDAC19 histone deacetylase-M64; MYC2 transcription factor-M70; and FUBP protein-M79), cellular transport and signal perception and transduction (OPT3 transporter-M19; CSC1 protein-M2A and M3A; mc410 protein from NINJA family-M53A), protein modification (F-box protein with LRR domain-M18), and other cellular processes, like photosynthesis (chlorophyll binding protein and LHC-I protein-M14, M15, M23, M24, M61, and M9), purine synthesis (adenylosuccinate synthetase-M49, M63, and M66) and cell detoxification (glutathione hydrolase-M71). Three dESTs encoded proteins involved in defense and plant-pathogen interactions: transcription factor RAP2-M2, EFR3 protein-M26, and ABH hydrolase-M75. Five dESTs (M12, M22, M51, M54, and M65) were overexpressed in infected tetraploid genotypes and showed no homology to known sequences stored in the National Centre for Biotechnology Information (NCBI) database.

### 2.7. Gene Expression Analysis

**Figure 9:**
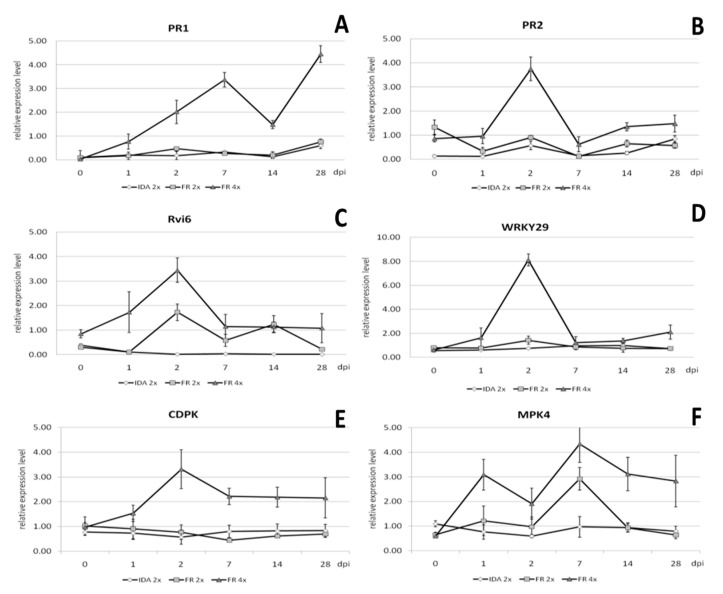
Relative gene expression analysis using real-time PCR of the genes (**A**) *PR1*, (**B**) *PR2*, (**C**) *Rvi6*, (**D**) *WRKY29*, (**E**) *CDPK*, and (**F**) *MPK4* genes in the tetraploid clones of the ‘apple ‘Free Redstar’ apple during *V. inaequalis* infection in relation to the diploid counterpart and ‘Idared’ reference susceptible to apple scab, analysed before (0) and 1, 2, 7, 14, and 28 days post inoculation (dpi). Error bars represent standard deviation.

Quantitative analysis of gene expression was performed in relation to the reference gene casein kinase II subunit beta-4 (*CKB4*), and the results are presented in Figure 9. The expression level of the *PR1* gene increased from 1 to 7 days post inoculation (dpi) and was 4.5-, 4.3-, and 12.5-fold higher, respectively, than in the diploid genotype (Figure 9A). The expression of this gene was highest on 28 dpi and was 6-fold higher than in the diploid genotype. Relative expression of the *PR2* gene was highest 2 dpi and was 4-fold higher than in the diploid genotype at the same time (Figure 9B). The increased level of *PR2* gene expression remained at a similar level from 14 to 28 dpi, being 2- and 2.5-fold higher compared to the diploid. 

Relative expression of the *Rvi6* gene specific to apple scab resistance in the tetraploid apple clones was also highest 2 dpi and was almost 2-fold higher than in the diploid (Figure 9C). On the 14 dpi, the expression of this gene remained similar in both tetraploid and diploid plants. The decrease in *Rvi6* expression was observed 28 dpi in diploid and was almost 5-fold lower compared to thetetraploid.

Relative expression of the *CDPK* gene associated with resistance to fungal diseases was the highest 2 dpi and was more than 4-fold higher than in the diploid (Figure 9E). From seven to 28 dpi, the relative level of expression of this gene in tetraploid plants remained at a similar level and was then almost 4-fold higher compared to diploid plants. Relative expression of the *MPK4* gene, also associated with resistance to fungal diseases, was highest on 7 dpi (Figure 9F). However, compared to the expression of this gene in diploid plants, it was only 1.5-fold higher. In the early stages of infection, 2 dpi, the relative expression of this gene was on average 2-fold higher than in diploid plants. After 14 and 28 dpi, an increased level of relative expression of the *MPK4* gene was also observed, and was 3.3- and 4.4-fold higher, respectively, compared to the expression of this gene in diploid plants on these days.

Of the tested genes, the highest relative expression was recorded for *WRKY29* in tetraploid plants on 2 dpi and was almost 6-fold higher compared to this gene expression in diploid plants at this time (Figure 9D). The *WKRY29* increased expression levels by 2–3 times compared to diploids and was observed one and 28 dpi.

## 3. Discussion

### 3.1. Morphological and Physiological Differences between Diploid and Tetraploid Plants

In our study, chromosome doubling caused a marked change in the phenotype of the studied apple ‘Free Redstar’ autotetraploids. Compared to diploid plants, the newly developed tetraploids had considerably shorter shoots, fewer branches and smaller stem diameters. The weak growth of own-rooted apple tetraploids was observed especially in young plants in the first growing season. In the third season, after the series of winter low temperature periods, the differences in tree height between the own-rooted diploid and tetraploid plants, although significant, were much smaller than those observed in the first season. Similar weak growth of neotetraploids in the first year of ex vitro cultivation was reported for daylily [36]. However, in the next season, following winter dormancy completion, daylily tetraploids were characterized by lush growth and larger leaves and flowers compared to diploids. The weaker growth of apple tetraploids observed in our research is probably related to the fact that they were still in the juvenile phase. Xue et al. [19] reported that autotetraploid apple plants showed slow growth and stunted features before reaching the generative phase, and their growth intensified after the first flowering. Another hypothesis referring to the explanation of dwarfism met in mitotic apple neotetraploids was reported by Ma et al. [37]. These authors found that the levels of hormones involved in cell expansion and elongation such as indoleacetic acid (IAA) and brassinosteroids (BRs) were significantly decreased in 3- and 5-year old autotetraploid plants. Gene expression analysis showed that the differentially expressed genes were mainly related to IAA and BR biosynthesis. 

We postulated that the weak growth of own-rooted apple tetraploids observed in young plants in the first season may have a transient character and we considered this issue later in the discussion in the context of changes in DNA methylation caused by genome duplication (see Section 3.2).

In addition, we observed that the leaves of apple tetraploids have an altered shape, they are more round. Similar changes in the leaf shape of apple tetraploids were previously reported by Sedysheva and Gorbacheva [16] and Xue et al. [19]. These authors also observed that the chlorophyll content, leaf thickness, and stomata size were increased in apple tetraploids compared to diploids, which is consistent with our results. A similar increase in chlorophyll content and stomata size are commonly observed in neotetraploids of various species, e.g., in mango [38] and daylily [36]. The strong positive correlation between stomata size and ploidy level is considered a morphological marker of ploidy level for many plant species, including apple [39,40]. 

In our study, physiological parameters, Pn, Tr, and WUE were similar in own-rooted diploids and tetraploidplants in the first growing season. In the third season, Tr, Ci, and Gs tended to be lower in tetraploids, whereas Pn and Φ_PSII_ were similar for both ploidy levels, and WUE tended to be higher in tetraploids compared to own-rooted diploid plants. WUE, i.e., water use efficiency, is defined as photosynthesized carbon per units of transpired water and is often used for estimation genotype tolerance to water deficit [41]. The higher WUE of tetraploids may indicate their higher tolerance to water stress, which was confirmed by Zhang et al. [42] and Hias et al. [21] on physiological and/or molecular levels. Under drought stress, ‘Hanfu’ and ‘Gala’ apple tetraploids had higher relative water content (RWC) and chlorophyll fluorescence parameters, while the lower levels of malondialdehyde (MDA) and proline, as well as lower expression levels of key aquaporin genes, *MdPIP1*;*1* and *MdTIP1*;*1*, indicated that tetraploids had enhanced drought tolerance compared to diploids [42]. Unlike in our study, Xue et al. [19] observed that all parameters of gas exchange and chlorophyll fluorescence were higher in apple tetraploids of the ‘Hanfu’ cultivar. These authors, however, performed measurements on mature plants that had already bloomed and, since that time, manifested increased vigour compared to their poor growth at the juvenile stage. 

In contrast to own-rooted trees, in M9-grafted plants, we did not observe any significant differences between diplo- and tetraploids, either in morphological or physiological parameters. In grafted plants, the only exceptions were the increased leaf thickness and CCI of tetraploids compared to diploid plants. In apple tree production, cultivar scions are grafted onto various types of rootstock that impart different properties, such as retarded growth, enhanced resistance to drought and diseases, or shortening the juvenile period (earlier flowering) [43]. The M9 dwarfing rootstock is most commonly used for grafting throughout the world. It was proposed that the dwarfing effect in the M9 interstem was initiated by inherently lower expression of *MdPIN8*, a gene related to auxin homeostasis, or by poor root zeatin synthesis in M9 [44]. Surprisingly in our studies, M9 reduced growth only of the diploid scion but this rootstock did not influence tetraploid growth since the vigour of both own-rooted and grafted tetraploids was similar. It was reported that the rootstock influences gene expression patterns in scions via various molecular mechanisms, depending on the scion genotype [45]. This may explain the varied impact of M9 rootstock on diploid and tetraploid scions of ‘Free Redstar’ apple.

### 3.2. Poor Vigor of Neotetraploids in the Context of Increased DNA Methylation

The reason for the poor growth of neotetraploids in the initial period of their growth and the supposed transient nature of this phenomenon is probably due to the much higher DNA methylation rate, recorded in six-month old tetraploid plants compared to the diploid donor plant. As in the current studies, our preliminary research also showed that the growth of apple tetraploids ‘Pinova’ and ‘Sander’ was very slow or almost completely inhibited; most of the plants manifested premature dormancy [23]. Those tetraploid plants were also characterized by a high DNA methylation rate that decreased following low-temperature treatment. We suspect that the process of entering dormancy and then resuming growth following low temperature treatment may be one of the factors involved in genome stabilization related to decreased DNA methylation. In contrast, He et al. [46] reported that DNA methylation rates, based on MSAP analysis, were very similar between diploid and tetraploid plants of ‘Hanfu’ apple, implying that chromosome doubling had no effect on the global level of DNA methylation. It was also confirmed by transcriptome analysis, which revealed that polyploidization had no discernible effect on the expression level of any of the DNA methylation-related genes, *MET1*, *DRM2*, and *CTM3*. However, the authors used for the analyses four-year old M26-grafted trees of an unknown ontogeny phase, juvenile or generative. In contrast, numerous reports on epigenetic changes in newly formed autotetraploids indicated that in many cases, the alteration of DNA methylation rates and patterns occur because of polyploidization [8,11]. In *Brassica rapa*, variations in cytosine methylation were a major consequence of chromosome doubling as detected in autotetraploids using MSAP analysis [47]. Similarly, increased cytosine methylation associated with enhanced accumulation of secondary metabolites were found in autopolyploids of *Cymbopogon* sp. by means of in situ immune detection of 5-methylocytosine sites [48]. Although many studies have been published on DNA methylation as the cause of epigenetic changes in neopolyploids, the persistence of these changes and the mechanism of their resolution are still not well understood. A planned comparative analysis of DNA methylation of apple diploids and their tetraploids of various cultivars in the juvenile and generative stages, of own-rooted and grafted trees will help to clarify the mechanism of this biological phenomenon.

### 3.3. Phenotypic and Genetic Variation among the Sibling Neotetraploids

In the current study, in addition to significant differences between diploid and tetraploid plants, we observed marked differences between sibling tetraploid clones 4x-1 and 4x-2 or 4x-3, particularly in leaf shape and some physiological parameters. At the same time, the AFLP analysis showed a genetic polymorphism among the tetraploid clones. The 4x-1 and 4x-5 tetraploids revealed higher polymorphism of about 4% compared to other tetraploid clones (4x-3 and 4x-4) with approx. 1% polymorphism, when evaluated in relation to diploids. This suggest that some phenotypic changes may be due to changes in DNA structure. It should be noted that the tetraploids 4x-1 and 4x-5, which were more genetically different from the diploids, were characterized by very poor growth and most of the plants died. This may indicate an unfavourable nature of the mutations that could occur as a result of polyploidization. In our preliminary studies, the genetic differences between apple diploids ‘Gala Must’, ‘Pinova’, and ‘Redchief’ and their neotetraploids were detected, based on AFLP analysis, to a similar range of 1.7–3.6% [23] as currently presented in ‘Free Redstar’. Our results referring to the genetic variation occurring in autotetraploids have been supported by numerous reports reviewed in several articles [8,12,15]. 

### 3.4. Presence of Rvi Genes in Diploid and Tetraploid Plants 

A number of studies have shown that induction of polyploidy is an effective way to increase resistance to fungal, bacterial, and viral pathogens in plants [10,14,49]. For example, neotetraploids of peanut revealed a higher level of resistance than diploids to fungal late leaf spot (LLS) caused by *Phaeoisariopsis personata* and viral disease *Peanut bud necrosis virus* (PBNV) [50]. Chromosome doubling was also an effective tool to obtain an *Anemone sylvestris* genotype tolerant to *Phytophthora plurivora* [51]. In our study, neotetraploids of ‘Free Redstar’ apple revealed a very high resistance level to apple scab, whereas its diploid counterpart was affected by this disease. Corresponding observations were reported for apple autotetraploids of ‘Hanfu’ and ‘Gala’ cultivars [20]. These neotetraploids were less susceptible to infection caused by fungal pathogens *Alternaria alternata* and *Colletotrichum gloeosporioides*, compared to diploid counterparts. The authors further confirmed that the higher resistance level was the result of increased expression levels of resistance-related genes recorded in tetraploids.

Our preliminary study, concerning one-year greenhouse testing for susceptibility to apple scab, showed that the tetraploids of the intermediate and high apple scab susceptibility, such as ‘Redchief’ and ‘Gala Must’, were characterized by similar susceptibility level as their diploid counterparts [23]. However, in the cultivars ‘Pinova’ and ‘Free Redstar’, bearing *Rvi5*, *Rvi6*, and *Rvi11* [52], most of their tetraploids were not affected or affected to a very small extent [23]. These results referring to ‘Free Redstar’ were further confirmed during the four-year studies presented here. Similar observations on apple tetraploids, which were originated from the diploid genotypes differing in susceptibility to apple scab, were reported by Hias et al. [22]. All this information indicates that more disease-resistant tetraploids are more likely to be obtained when more resistance-related genes are available in the diploid progenitor.

Resistance to apple scab has quantitative and qualitative characteristics [1]. Our analysis of apple scab resistance genes confirmed the presence of six of the eight *Rvi* genes analysed in ‘Free Redstar’ apple tetraploids and its diploid counterpart. We observed PCR amplicons specific for *Rvi5*, *Rvi6*, and *Rvi11*, as previously confirmed by Korbin et al. [52], as well as other genes, *Rvi8*, *Rvi14*, and *Rvi17*, identified in ‘Free Redstar’ for the first time. In the case of the *Rvi6* region amplification with ACS9 and *Rvi11* region with T06 primers, an increased amount (intense bands) of specific PCR products were recorded in tetraploid clones compared to the diploid counterpart. This indicates the multiplication of resistance genes in these genotypes and lack of mutation in these DNA fragments because of polyploidization. The presence of a 740 bp product amplified with the K08 primer, which is a fragment of the *Rvi11* gene, was previously confirmed by Korbin et al. [52] in apple cultivars known to be relatively tolerant to apple scab, including ‘Free Redstar’. In our study, we observed an increased amount of the 740 bp product in tetraploids and a reduced amount of the 900 bp product compared to the diploid genotype. The product of 900 bp is probably the second locus of the *Rvi11* gene [52]. In the reaction with T06 primers specific to the *Rvi11* gene, none of the studied genotypes produced a 400 bp product, which is probably a fragment of this gene [53]; only the 800 bp fragment was observed. Like Korbin et al. [52], we did not confirm the presence of *Rvi7* and *Rvi15* in the tetraploid ‘Free Redstar’ apple. Difficulty in interpreting the results of *Rvi* genes’ analysis in ‘Free Redstar’ is due to the unknown origin of this cultivar. It was selected from progeny derived from seeds obtained from the USA [54]. The results of molecular analyses indicate that ‘Free Redstar’ bears at least six pyramided *Rvi* genes. Based on the knowledge of the pedigree of the hosts of the genes for resistance to apple scab, described in detail by Bus et al. [1], it can be concluded that wild *Malus* species and old cultivars are among the progenitors of ‘Free Redstar’: *M. floribunda* 821 (*Rvi6*), *M. baccata jackii* (*Rvi 6*, *Rvi11*), *M. micromalus* (*Rvi5*), *M. sieviersii* (*Rvi8*), and ‘Dülmener Rosenapfel’ (*Rvi14*), a scab-resistant cultivar raised from open-pollinated ‘Gravenstein’ and Antonovka APF22 (*Rvi17*). In summary, as a result of polyploidization, the *Rvi* genes in ‘Free Redstar’ cultivar were multiplied in its tetraploids, which contributed to an increase of their resistance to apple scab.

### 3.5. Enhanced Resistance of Neotetraploids to Apple Scab as the Result of Increased Expression Levels of Resistance-Related Genes 

Very high resistance to apple scab found in ‘Free Redstar’ neotetraploids was also confirmed by our results of the quantitative analysis of the expression of several resistance-related genes, which was performed under *V. inaequalis* infection compared to diploids and the ‘Idared’ susceptible reference. Different expression profiles were observed for the analysed genes. This indicated that the activity of the proteins encoded by these genes occur at different stages of resistance reactions. We found that the activities of four genes (*PR2*, *Rvi6*, *WRKY29*, and *CDPK*) were the highest in tetraploids at 2 dpi and these activities were much higher than in the diploid ‘Free Redstar’ and ‘Idared’ reference. Furthermore, the expression levels dropped down and remained constant but still higher than in diploids and the reference. In turn, in tetraploid ‘Free Redstar’, activity peaks were observed at 7 and 20 dpi for *PR1*, and at 1 and 7 dpi for *MPK4*. In the diploid ‘Free Redstar’, an increased activity in relation to ‘Idared’ was observed only for the *Rvi6* and *MPK4* genes but was lower than in tetraploids. Similar tendencies in gene expressions were reported by Chen et al. [20]. They showed that during infection of ‘Gala’ and ‘Hanfu’ apple cultivars with *Alternaria alternata* and *Colletotrichum gloeosporioides*, *PR1*, *WRKY29*, *CDPK*, and *MPK4* genes were upregulated, either in diploid or tetraploid plants, but the expression levels of these genes were much higher in tetraploids. Overexpression of *PR* genes, individually or collectively, caused the strengthening of defense reactions in plants against a wide range of pathogens [31]. Our thesis on the multiplication of apple scab resistance genes in tetraploid genotypes was confirmed by Hias et al. [22], who showed that the apple neotetraploid genotypes (G58) containing the *Rvi6* locus revealed significantly increased resistance to *V. inaequalis*. However, the enhanced expression of resistance gene does not always translate into enhanced resistance to the pathogen as it was reported for *Rvi6* gene expression in genotypes differing in resistance to apple scab [55].

Based on our results of transcriptome analysis, it can be concluded that systemic acquired resistance (SAR) related to the presence of PR (pathogenesis related) proteins [31] determines the defense responses of ‘Free Redstar’ tetraploids against fungal disease. PR antifungal proteins, including PR1 and PR2, belong to a group of proteins which are activated during a plant defense response to pathogen attack. Thus, expressions of these genes and other genes associated with immune responses such as *WRKY29*, *CDPK*, and *MPK4* increase as has been observed in ‘Free Redstar’ apple tetraploids inoculated with *V. inaequalis*. Similarly in *Brassica napus*, changes in the expression of genes encoding proteins of the WRKY family were observed during infection with *Sclerotinia sclerotiorum* and *Alternaria brassicae* [56]. For the majority of this family’s genes, the increased expression was observed following the pathogen infection. Furthermore, the relationship between the expression of *WRKY* genes and the synthesis of jasmonic acid (JA), salicylic acid (SA) and ethylene (ET) was recorded. SA as a signal molecule (messenger) is associated with SAR plant immune system, while JA and ET are involved in an induced systemic resistance (ISR) defense mechanism [57,58]. Overexpression of the *WRKY65* gene was reported for *Paeonia lactiflora* during infection by *Alternaria tenuissima* [59]. As in the apple ‘Free Redstar’ tetraploids inoculated with *V. inaequalis*, expression of *PlWRKY65* in *P. lactiflora* was increased sharply and peaked 24 h after inoculation with *A. tenuissima*, and was 16-fold higher than in the non-inoculated plants. Moreover, it was found that the gene-silenced *P. lactiflora* plants were more sensitive to *A. tenuissima* infection than the wild plants. Other resistance-related proteins, CDPKs are important components of the plant immune response. Acting with the mitogen activated protein kinases (MAPK/MPK), they positively regulate defense gene expression upon bacterial infection [60]. Overexpression of *OsCPK4* in *Orysa sativa* leads to an enhanced disease resistance against *Magnaporthe oryzae*. In *Arabidopsis*, MPK4 protein constitutes a signaling cascade and interacts with WRKY33, which in turn regulates the expression of further genes encoding proteins involved in resistance response [61]. The interaction of the MPK4 and WRKY proteins may explain the changes in their gene expressions that we observed in tetraploid plants infected with *V. inaequalis*. In *Arabidopsis*, MAPK cascades positively regulate responses against the bacterial pathogen *Pseudomonas syringae* pv. *tomato* and the fungal pathogen *Botrytis cinerea* [62]. 

### 3.6. Comparative Transcriptome Analysis of Diploid and Tetraploid Apple Plants Inoculated with V. inaequalis

Continuing the studies referring to the genetic background of enhanced tetraploid resistance to apple scab, we analysed, using the cDNA-AFLP method, the transcriptomes of the three tetraploid clones in relation to the diploid counterparts 48 h after inoculation with *V. inaequalis*. Using cDNA-AFLP analysis, we identified ESTs fragments homologous to genes encoding known plant proteins involved in gene expression regulation, cell transport and signal transduction, post-translational protein modification, and other cellular processes. Among the genes encoding proteins involved in plant immune responses, we identified three genes encoding transcription factor RAP2, the EFR3 protein, and ABH hydrolase. The RAP2 protein belongs to the ethylene-dependent family of transcription factors. It probably acts as a transcription activator and may be involved in the regulation of gene expression in signalling pathways triggered in stressful conditions, although the main function of this type of protein in plant cells is response to oxygen deficiency [63]. The EFR3 protein is a serine-threonine kinase involved in the regulation of gene expression and cell signal transduction. It acts as a PPR (pattern-recognition receptor), recognizing pathogen *Avr* genes and inducing a strong defense response [64]. Both abiotic and biotic factors can cause overexpression of hydrolases in plant cells; ABH hydrolase functions in plant cells as a ligand for phytohormone receptors in signalling pathways, e.g., gibberellins [65]. 

We also detected some other genes that were upregulated under *V. inaequalis* infection that are involved in plant resistance responses. Histone deacetylase HDA19 as a transcription regulator (repression) at the chromatin level related to cell signalling involving JA and ET in the response to pathogens [66]. The MYC2 transcription factor belonging to the family of the bHLH (basic-helix-loop-helix) factors type is a transcription activator associated mainly with ABA and JA signalling pathways. It has been shown that the gene encoding this transcription factor is overexpressed during the response to pest and pathogen infection in *A. thaliana* [67]. The FUBP (far upstream element-binding protein), in turn, is a protein that regulates gene expression by binding RNA. It has been shown that it can regulate expression by interacting with MYC-type transcription factors. During the infection of apple with *V. inaequalis*, genes encoding transport proteins and proteins responsible for signal transduction were also overexpressed. Transporter OPT3 (oligopeptide transporter 3) belongs to the OPT protein family, which together with the ABC and PTR transporters family, is the most widespread family of proteins, including plant transporters, and acts as a transmembrane transporter of metal ions, as well as tetra- and pentapeptides. Although the function of these transporters is not fully described, it is suspected that they may be involved in the transport of important regulatory molecules [68]. The CSC1 protein also belongs to the transmembrane proteins included in Ca^2+^ ion transport protein complexes. Recent studies indicated that these proteins convert mechanical stimuli reaching the cell into a cascade of signals involving ions [69]. In turn, the mc410 protein from the NINJA (novel interaction of JAZ-jasmonate ZIM-domain repressor protein) family plays an important role in signalling cascades related to the cell’s response to stress and function as a JA-associated transcriptional repressor regulating many signalling pathways through interaction with specific adaptive proteins [70]. We also identified a gene encoding a protein involved in post-translational protein modifications. The F-box protein with the LRR (leucine-rich repeat) domain takes part in the ubiquitisation of proteins by regulating cellular activity at the protein level (degradation of incorrectly synthesized, incorrectly distributed, or aging proteins). It was reported that F-box proteins with an LRR domain may be involved in pathogen recognition and accumulation of NLR (nucleotide-binding leucine-rich repeat-type) receptors, transport of proteins, and transcription factors [71]. 

The identified dESTs and the analyzed genes encoding known proteins related to resistance to infection with the fungal pathogen show a relationship with both SA and JA/ET involved in SAR and ISR pathways, respectively. This means that the plant activates two types of induced resistance, both SAR and ISR. Ji et al. [72], analyzing *Brassica rapa* transcriptomes during *Plasmodiophora brassicae* infection, suggested that these two resistance mechanisms show some degree of interaction and may occur simultaneously. It is possible, that similar immune system against *V. inaequalis*, including SAR and ISR pathways, exist both in diploid and tetraploid apple plants of ‘Free Redstar’, however, in the latter, defense mechanism is significantly enhanced due to a double dose of resistance genes.

### 3.7. Summary

Molecular analysis showed that ‘Free Redstar’ cultivar bears six *Rvi* genes. Three of them, *Rvi5*, *Rvi11*, and *Rvi14* are considered to confer durable apple scab resistance and *Rvi6* has been estimated to be useful in resistance breeding as well, since its resistance is overcome by the pathogen with medium frequency [4]. The activities of various genes related to resistance were strongly enhanced in tetraploids of the ‘Free Redstar’ apple cultivar, as evidenced by their significantly increased expression levels compared to their diploid counterparts. Based on transcriptome analysis, several other genes related to plant resistance responses were found to be upregulated. Hence, the tetraploids showed almost no disease symptoms under provoking (high humidity) conditions, while their diploid counterparts were affected by *V. inaequalis*. Moreover, our results suggest that the poor vigour of neotetraploids in the early period of their growth in the juvenile phase may have a transient character related to their higher degree of DNA methylation. However, many questions remain: what is the behaviour of the remaining *Rvi* genes and other genes that are related to biotic stress resistance and tolerance to abiotic stress; what are the differences in DNA methylation rate and pattern between the own-rooted and grafted trees or between the plants at juvenile and generative phases? We expect that the obtained apple tetraploids will bloom in 2021, and then it will be possible to conduct further molecular analyzes. Continuing research, we may further elucidate the mysteries of the phenomenon of phenotypic and genetic changes accompanying the process of polyploidization.

The results obtained are the basis for developing further research on the more in-depth assessment of tetraploids of several apple cultivars, as well as to evaluate the usefulness of tetraploids for breeding triploid cultivars with increased resistance to diseases and drought.

## 4. Materials and Methods

### 4.1. Plant Material

The diploid ‘Free Redstar’ apple cultivar and its synthetic autotetraploids were used for this study. ‘Free Redstar’ is a cultivar of unknown pedigree; the seedling was selected from a progeny derived from seeds received from the USA—the seeds were obtained in a frame of the PRI cooperative scab-resistance apple breeding program between Purdue University, Rutgers, The state University of New Jersey and the University of Illinois, USA [73]. Fruits, formed mainly on spurs and shoot tips, are oblong-conical, with small ribs around the calyx. It ripens just after ‘Freedom’. This cultivar possesses a relatively high level of resistance to apple scab determined by three genes *Rvi5*, *Rvi6*, and *Rvi11* [52], powdery mildew, and fire blight. Autotetraploid clones of ‘Free Redstar’ apple were obtained using in vitro techniques [25]. Selected tetraploids were cloned to 20–30 plants and rooted and grown in a greenhouse [26]. Plants of one to five tetraploid clones, 4x-1, 4x-2, 4x-3, 4x-4, and 4x-5 were evaluated in terms of phenotype and/or genotype in relation to their diploid counterparts. Due to the limited number of tetraploid plants of the clones: 4x-1, 4x-4, and 4x-5, caused by their poor growth (some plants died out), these clones were used only for some measurements and analyses, when stated. Depending on the evaluation term and type, the plant material for examination was derived directly from in vitro culture or there were six- to eight-month old own-rooted plants grown in a greenhouse, as well as three-year old plants, both own-rooted and grafted on M9 rootstock, grown in the experimental orchard of the Research Institute of Horticulture, Skierniewice, Poland.

### 4.2. Morphological Observation 

Measurements included the height and diameter of the shoots, leaf size, and stomata size. Six leaves from each of five plants of each genotype and tree type (own-rooted and grafted) were measured for leaf area, length, and width using a planimeter. Six-month old own-rooted plants and three-year old own-rooted and grafted trees were observed.

The size of stomata were measured under an Eclipse-80i light microscope (Nikon, Tokyo, Japan) using an image analysis system (NIS-Elements Basic Research) on microscopic preparations made using isolated epidermis fragments picked from a leaf using transparent Scotch-type adhesive tape and dyed with 2% toluidine blue. For each genotype, the length of stomata was measured for 10 leaves (×10 stomata). Microscopic photographic documentation was made using a CCD PS-Fi1 monochrome digital camera (Nikon, Tokyo, Japan) at 400× magnification. 

### 4.3. Evaluation of Physiological Parameters

The relative chlorophyll content index (CCI), gas exchange, and chlorophyll fluorescence were evaluated in six-month old own-rooted plants and three-year old trees, both own-rooted and grafted. From four to five plants of each genotype (diploid and tetraploid clones) were evaluated. Relative chlorophyll content was measured with a CCM-200 Chlorophyll content meter (Opti-Sciences Int., Hudson, NH, USA). Gas exchange was measured using a Portable Photosynthesis System (LI-COR Biosciences GmbH, Bad Homburg, Germany). Measurements included net photosynthetic rate (Pn), internal CO_2_ concentration (Ci), stomatal conductance (Gs), and transpiration rate (Tr) and were performed on sunny days at the end of July from 11 a.m. and 2 p.m., under a photosynthetic photon flux density of approximately 1200 µmol m^−2^ s^−1^. Water use efficiency (WUE) was calculated as a ratio between Pn and Tr (Pn/Tr). Fluorescence of chlorophyll was measured using a fluorometer MINI PAM (Walz, Fehraltorf, Switzerland). The measurements included quantum efficiency of open photosystem II centres (Fv/Fm), and photosynthetic quantum yield (Φ_PSII_).

### 4.4. Testing for Susceptibility to Apple Scab

The assessment was performed in a greenhouse on eight-month old own-rooted plants. They were inoculated with a spore suspension of *Venturia inaequalis* with a concentration of about 10^5^ spores/mL, obtained as a result of washing with water the spores from heavily infected apple leaves taken from the RIH experimental orchard (organic plantings). After inoculation, the plants were placed under favourable conditions for infection (98–100% relative humidity and 20–25 °C) for 48 h. After this time, the plants were transferred to standard greenhouse conditions (temperature 18–25 °C). Leaf infection assessment was carried out four weeks after inoculation using a six-point scale, in which 0 was 0%, 1 was 0.5%, 2 was 3.0%, 3 was 12.5%, 4 was 35.0%, and 5 was 75.0% of the leaf area affected by the fungus. The three youngest fully developed leaves of three plants of each genotype were observed. The means of each plant were taken for analysis of variance. The assessment of leaf infection degree was repeated in the four consecutive years of the study.

### 4.5. AFLP Analysis

The genetic evaluation of four ‘Free Redstar’ autotetraploids (4x-1, 4x-2, 4x-3, 4x-5) in relation to their diploid counterpart (2x) were carried out using the AFLP method. DNA was extracted from fresh plant tissue randomly selected from the own-rooted six-month old plants of each tetraploid clone growing in a greenhouse and from donor diploid plants growing in the experimental orchard. Genomic DNA was extracted using the DNeasy Plant Mini Kit (Qiagen, Hilden, Germany) in three replicates for each sample tested. The concentration and purity of the DNA were determined using an Epoch spectrophotometer (BioTek, Highland Park, VT, USA). Five AFLP primer pairs were finally used in the study after an initial screening of 10 AFLP primer pairs for the production a high number of distinct and scorable bands (Table S1). 

For AFLP analysis, genomic DNA (50 ng) was digested with *Mse*I and *Pst*I endonucleases and ligated with appropriate adapters [74]. Preamplification of obtained fragments was carried out using primers complementary to the adapter’s sequence. The preselection PCR reaction mixture containing 20 μL DNA, 1x Taq Polymerase Reaction Buffer (Sigma-Aldrich, Steinheim, Germany), 0.8 μL dNTPs (10 mM) (Promega, Madison, WI, USA), 1.2 μL of each primer (10 μM), and 0.75 U Taq DNA Polymerase (Sigma-Aldrich, Steinheim, Germany). Amplification was carried out in the thermal cycler programmed for 30 cycles (30 s at 94 °C, 30 s at 60 °C, 60 s at 72 °C). Selective PCR with primer pairs obtained by extending the primers were used in preamplification with two additional nucleotides at the 3′end. PCR was carried out in the reaction mixture containing 3 μL DNA 1:30 (*v*:*v*), 1 × Taq Polymerase Reaction Buffer (Sigma-Aldrich, Steinheim, Germany), 0.4 μL dNTPs (10 mM) (Promega, Madison, WI, USA), 0.5 μL *Pst*I-NNprimer (10 μM), 0.65 μL *Mse*I-NN primer (10 μM), and 0.75 U Taq DNA Polymerase (Sigma-Aldrich, Steinheim, Germany). Amplification was carried out in the thermal cycler programmed for 13 cycles (30 s at 94 °C, 30 s at 65–56 °C with annealing temperature decreased by 0.7 °C in each cycle, 60 s at 72 °C), followed by 26 cycles (30 s at 94 °C, 30 s at 56 °C, 60 s at 72 °C). Products of selective PCR were separated on 6% denaturing polyacrylamide gel through electrophoresis on Dual Dedicated Height Nucleic Acid Sequencer (C.B.S. Scientific, San Diego, CA, USA). The separated AFLP products were stained in a silver nitrate solution, and the gel was dried, described, and photographed. The size of the bands was assessed against size standards of a 10 bp DNA Ladder and 50 bp DNA Ladder (Invitrogen by Thermo Fisher Scientific, Waltham, MA, USA). Generated bands were scored manually as present (1) or absent (0) from the photographs. Only bright and reproducible products were scored. During the analysis of electrophoregrams, the number of AFLP products, their size, and diversity between mother diploid plants and tetraploid plants were evaluated.

### 4.6. Methylation-Sensitive Amplification Polymorphism (MSAP) Analysis

Possible epigenetic changes occurring in the genome of two tetraploids (4x-2 and 4x-3) and diploid counterparts (2x) were analysed using the MSAP method according to Xiong et al. [75], Peraza-Echeverria et al. [76], and Fulneček and Kowařik [76,77]. DNA was extracted from fresh plant tissue collected from randomly selected in vitro plantlets of each tetraploid clone, as well as from six-month old plants growing in a greenhouse and diploid donor plants growing in the experimental orchard. Genomic DNA was extracted as described above. 

For MSAP analysis, genomic DNA (50 ng) was digested with restriction enzymes *Eco* R1, *Msp*1, and *Hpa*11 in three different combinations and ligated with appropriate adapters. Preamplification of obtained fragments was carried out using primers complementary to the adapter’s sequence. The preselection PCR reaction mixture contained 3.0 μL DNA, 1x Taq polymerase reaction buffer (Sigma-Aldrich, Steinheim, Germany), 0.8 μL dNTPs (10 mM) (Promega, Madison, WI, USA), 1.2 μL of each primer (10 μM), and 0.75 U Taq DNA Polymerase (Sigma-Aldrich, Steinheim, Germany). Amplification was carried out in the thermal cycler programmed for 30 cycles (30 s at 94 °C, 30 s at 60 °C, 60 s at 72 °C). Selective PCR with six primer pairs were obtained by extending the primers used in preamplification with two additional nucleotides at the 3′end (Table S1). PCR was carried out in the reaction mixture containing 3 μL DNA 1:10 (*v*:*v*), 1x Taq polymerase reaction buffer (Sigma-Aldrich, Steinheim, Germany), 0.4 μL dNTPs (10 mM) (Promega, Madison, WI, USA), 0.5 μL E-NN primer (10 μM), 0.65 μL MH-NN primer (10 μM), and 0.75 U Taq DNA Polymerase (Sigma-Aldrich, Steinheim, Germany). Amplification was carried out in the thermal cycler programmed for 13 cycles (30 s at 94 °C, 30 s at 65–56 °C with annealing temperature decreased by 0.7 °C in each cycle, 60 s at 72 °C), followed by 26 cycles (30 s at 94 °C, 30 s at 56 °C, 60 s at 72 °C). Products of selective PCR were analysed as described above. The results of MSAP analyses were interpreted in accordance with Fulneček and Kowařik [77]. 

### 4.7. Determination of Rvi Genes

The experiments were undertaken on three tetraploid clones (4x-1, 4x-2, and 4x-3) and their diploid counterparts (2x). DNA was extracted from fresh plant tissue of young leaves collected from six-month old randomly selected plants. Genomic DNA was extracted using the DNeasy Plant Mini Kit (Qiagen, Hilden, Germany) in three replicates for each sample tested. The concentration and purity of the DNA were determined using an Epoch spectrophotometer (BioTek, Highland Park, VT, USA). 

Analysis assessed the presence of the eight resistance genes: *Rvi5*, *Rvi6*, *Rvi7*, *Rvi8*, *Rvi11*, *Rvi14*, *Rvi15*, and *Rvi17*. Gene expression analysis was performed using the PCR technique. As a reference susceptible to apple scab, the ‘Idared’ cultivar was used. PCR reactions were performed with specific primers designed according to the reviewed data [5,52]. The primer sequences are presented in Table S1.

PCR was carried out in a reaction mixture containing 20 ng DNA, 1X Taq polymerase reaction buffer with 10 mM MgCl_2_ (Sigma-Aldrich, Steinheim, Germany), 0.4 μL dNTPs (10 mM) (Promega, Madison, WI, USA), 0.5 mM of each primer, and 5 U Taq DNA Polymerase (Sigma-Aldrich, Steinheim, Germany). Amplification was carried out in a T100 Thermal Cycler (Bio-Rad, Hercules, CA, USA) programmed for 35 cycles (30 s at 94 °C, 30 s at 58–67 °C depending on the primers, 60 s at 72 °C). Products of selective PCR were separated on 1% agarose gel with Simply Safe^TM^ Dye (EURx, Gdansk, Poland) through electrophoresis in Multi Sub^TM^Screen (Clever Scientific Ltd., Rugby, Warwickshire, United Kingdom) and photographed using Syngen Biotech camera (Wroclaw, Poland). The size of the bands was assessed against size standards GeneRuler^TM^ 100 bp DNA Ladder Plus (Thermo Fischer Scientific, Waltham, MA, USA). Bands generated in PCR analysis were scored manually as present (1) or absent (0) from the photographs. Only bright and reproducible products were scored. During the analysis of electrophoregrams, the number of PCR products, their size, and diversity between tetraploid plants and diploid plants and sensitive standard were evaluated.

### 4.8. Analysis of Transcriptome and Expression of Resistance-Related Genes under V. inaequalis Infection

#### 4.8.1. Transcriptome Analysis (cDNA-AFLP Analysis)

The experiments were undertaken on three tetraploid clones (4x-1, 4x-2, and 4x-3) in comparison to their diploid counterparts (2x). The tissue samples of leaves for molecular analyses were collected before inoculation (control sample) and 48 h after *V. inaequalis* inoculation as described in Section 4.4. Plant material was put into liquid nitrogen and stored at −80 °C until analyses.

To identify genes whose expression was modulated by *V. inaequalis* infection, a cDNA-AFLP technique was used according to Money et al. [78]. The tissue samples were ground in liquid nitrogen and total RNA was extracted using a RNeasy Plant Mini Kit (Qiagen, Hilden, Germany). The RNA extract was treated with DNase RQ (Promega, Madison, WI, USA) to remove any traces of DNA. mRNA was isolated using Oligotex mRNA Kit (Qiagen, Hilden, Germany) and transcribed to cDNA using M-MLV Reverse Transcriptase (Promega, Madison, WI, USA). Further analyses with obtained cDNA were performed according to the protocol described in Section 4.5 with 10 AFLP primer pairs (Table S1). During analysis of polyacrylamide gels, only the amplicons that were qualitatively differentially displayed in tetraploid genotypes compared to diploid ones were selected for further analysis. Amplified cDNA fragments with a differential expression pattern (differentially Expressed Sequence Tags, dEST) were re-amplified from polyacrylamide gels and sequenced (Genomed, Warsaw, Poland). Sequences of cDNA fragments were subjected to analysis with the Basic Local Alignment Search Tool (BLAST) in the National Centre for Biotechnology Information (NCBI) database.

#### 4.8.2. Gene Expression Analysis

Expression analysis of selected resistance-related genes was performed for one tetraploid clone (4x-2) and diploid (2x) during *V. inaequalis* infection. The ‘Idared’ cultivar was used as a reference susceptible to apple scab. For analysis, young leaf samples were collected from six-month old plants 1, 2, 7, 14, and 28 days post inoculation(dpi) with the pathogen (see Section 4.4). The control samples were collected before inoculation (0 day). Leaves were put directly into liquid nitrogen, ground in liquid nitrogen, and stored at −80 °C. Total RNA was extracted using a RNeasy Plant Mini Kit (Qiagen, Hilden, Germany) from pooled plant tissue samples in three replications. Each RNA sample was digested with RQ RNase-Free DNase (Promega, Madison, WI, USA) and purified from the reaction mixture using a RNeasy Mini Kit (Qiagen, Hilden, Germany) according to the manufacturer’s instructions. Quantitative and qualitative analysis of RNA was examined using an Epoch spectrophotometer (BioTek, Highland Park, VT, USA). From each sample, 1 µg RNA was transcribed using M-MLV reverse transcriptase (Promega, Madison, WI, USA) and oligo(dT)15 primer (Promega, Madison, WI, USA). The cDNA samples obtained were used in gene expression analyses.

The analysis assessed the expression of the six genes: *PR1*, *PR2*, *Rvi6*, *WRKY29*, *CDPK*, and *MPK4*. Gene expression analysis was performed using quantitative real-time PCR. As a reference gene, the constitutively expressed *CKB4* (Casein kinase II subunit beta-4) gene was applied [79]. PCR reactions were performed with specific primers designed according to the literature [20]. The sequences of primers are shown in Table S1. Quantitative reverse transcription PCR (RT-qPCR) was undertaken with the KAPA^TM^Sybr Fast qPCR Master Mix (Kapa Biosystems, Amsterdam, The Netherlands) according with manufacturer’s instruction, with the annealing temperature for all primers 60 °C in a Rotor-Gene 6000 machine (Corbett Research, Bath, United Kingdom). All PCR reactions were undertaken with three technical replicates, and at the end of each PCR reaction, the melting curves of the amplified products were analysed (temperatures from 72 to 95 °C with raised by 1 °C/5 s). Additional, four 10-fold dilutions of cDNA were run together with analysed samples for the PCR efficiency calculation and generation of the standard curve (with correlation coefficient >0.99). Amplification products were analysed additionally by electrophoresis and sequenced to confirm their homology to the original sequences. For relative quantification, the standard curve method was applied [80] and the relative mRNA level of tested gene was normalized to the reference. The data were analysed using Rotor-Gene 6000 Series Software 1.7 (Corbett Research, Bath, United Kingdom).

### 4.9. Statistical Analyses

The results were subjected to statistical analysis with Statistica 10. Analysis of variance was performed, and the means compared using Duncan’s test (*p* = 0.05).

## Figures and Tables

**Table 1:** Morphological and physiological differences between diploid and tetraploid six-month old own-rooted plants of ‘Free Redstar’ apple.

Trait	Diploid	Tetraploid *	*p*
Shoot length (cm)	54.1 a	14.5 b	0.000
Stem diameter measured at the middle of the shoot (mm)	4.9 a	4.1 b	0.005
Number of nodes	27.0 a	16.7 b	0.000
Leaf area (cm^2^)	39.1a	31.6 a	0.100
Leaf length (cm)	9.7 a	7.7 b	0.005
Leaf width (cm)	5.7 a	5.9 a	0.503
Leaf length/width	1.71 a	1.30 b	0.000
Stomata length (µm)	28.3 b	37.8 a	0.000
Chlorophyll content index (CCI)	32.0 b	38.4 a	0.000
Net photosynthesis rate (Pn) (µmol CO_2_ m^−2^ s^−1^)	9.94 a	8.68 a	0.276
Transpiration rate (Tr) (mmol m^−2^ s^−1^)	2.73 a	2.45 a	0.132
Water-use efficiency (WUE)	3.63 a	3.59 a	0.916
Quantum efficiency of open photosystem II centres (Fv/Fm)	0.797 a	0.768 b	0.024
Photosynthetic quantum yield (Φ_PSII_)	0.154 b	0.200 a	0.019

* Values in column of tetraploid plants are the averages of the three tetraploid clones, 4x-1, 4x-2, and 4x-3. Means for each trait marked with the same letter do not differ significantly at *p* = 0.05; Duncan’s test.

**Table 2:** Rate of *Venturia inaequalis* infection of apple tetraploid ‘Free Redstar’ leaves in relation to their diploid counterpart (2x) and to the reference cultivar ‘Idared’ in a greenhouse trial. Fungal lesion was assessed using a six-point scale, in which 0 was 0%, 1 was 0.5%, 2 was 3.0%, 3 was 12.5%, 4 was 35.0%, and 5 was 75.0% of the leaf area affected by the fungus.

Genotype	Fungal Lesion (Scale 0–5)			
	2017	2018	2019	2020
‘Idared’ (reference)	4.0 a	3.3 a	-	2.0 a
‘Free Redstar’ 2x	1.5 b	3.7 a	4.3 a	2.7 a
4x-1	0.0 c	0.0 b	0.0 b	0.0 b
4x-2	0.0 c	0.0 b	0.1 b	0.0 b
4x-3	0.0 c	0.0 b	0.1 b	0.0 b
4x-4	0.0 c	0.0 b	0.0 b	0.0 b

a, b, c, means within columns marked with the same letter do not differ significantly at *p* = 0.05 according to Duncan’s test.

**Table 3:** Analysis of the presence of *Rvi* genes in tetraploid genotypes (4x-1, 4x-2, 4x-3) of ‘Free Redstar’ apple compared to the diploid (2x) counterpart and ‘Idared’ reference sensitive to apple scab.

*Rvi* Genes	Genotypes				
	4x-1	4x-2	4x-3	2x	Idared 2x (Reference)
*Rvi5*	+	+	+	+	−
*Rvi6*	+	+	+	+	−
*Rvi7*	−	−	−	−	−
*Rvi8*	+	+	+	+	−
*Rvi11*	+	+	+	+	−
*Rvi14*	+	+	+	+	−
*Rvi15*	−	−	−	−	−
*Rvi17*	+	+	+	+	−

+ Polymerase chain reaction (PCR) products associated with resistance to apple scab; − no PCR products or non-specific PCR products.

**Table 4:** Homology and functional classification of upregulated dESTs during *V. inaequalis* infection of ‘Free Redstar’ tetraploid genotypes.

EST ID	Basic Local Alignment Search Tool (BLAST) Annotation	Gen Bank Access Code	*E* Value	Functional Classification
	Homology to Genes from NIH GenBank			
M12	No homology			
M14	Chlorophyll a-b binding protein 6 (*Malus* × *domestica*)	XP_028961659.1	8e^−83^	other (photosynthesis)
M15	Chlorophyll a-b binding protein 6 (*Prunus* × *avium*)	XP_021832771.1	3e^−88^	other (photosynthesis)
M17	Hypothetical protein DVH24_023262 (*Malus × domestica*)	RXI09118.1	2e^−88^	unknown
M18	Putative F-box/LRR-repeat protein (*Brassica* × *rapa*)	XP_009131094.1	2e^−18^	protein modification
M19	oligopeptide transporter 3-like isoform X2 (*Malus × domestica*)	XP_028963015.1	2e^−05^	cellular transport
M21	Hypothetical protein (*Malus × domestica*)	RXI09118	2e^−87^	unknown
M22	No homology			
M23	Chlorophyll a-b binding protein (*Pyrus × bretschneideri*)	KJ008954.1	3e^−178^	other (photosynthesis)
M24	Chlorophyll a-b binding protein 6 (*Prunus* × *persica*)	XM_007201177.2	2e^−179^	other (photosynthesis)
M25	Uncharacterized protein (*Malus × domestica*)	XM_008372153.3	6e^−88^	unknown
M26	EFR3 protein (*Juglans* × *regia*)	XM_018960757.1	1e^−14^	defense and host-pathogen interaction
M2A	CSC1-like protein HYP1 isoform X1 (*Malus × domestica*)	XM_008343386.3	3e^−143^	cellular transport, signal transduction
M2	Ethylene-responsive transcription factor RAP2-7 isoform X2 (*Malus × domestica*)	XP_028944297.1	5e^−17^	defense and host-pathogen interaction, signal transduction
M3A	CSC1-like protein HYP1 isoform X1 (*Malus × domestica*)	XP_008341608.1	2e^−51^	cellular transport signal transduction
M49	Adenylosuccinatesynthetase 2 (*Malus × domestica*)	XM_008384161.3	2e^−89^	other (purine synthesis)
M51	No homology			
M53A	Ninja-family protein mc410-like (*Malus* × *domestica*)	XM_029110229.1	3e^−106^	signal transduction
M54	No homology			
M61	Chlorophyll a-b binding protein 151 (*Malus × domestica*)	XM_008381765.3	1e^−46^	other (photosynthesis)
M63	Adenylosuccinatesynthetase 2 (*Pyrus × bretschneideri*)	XM_009380296.2	2e^−94^	other (purine synthesis)
M64	Histone deacetylase 19-like protein (*Malus × domestica*)	XM_008350733.3	1e^−09^	gene expression regulation
M65	No homology			
M66	Adenylosuccinatesynthetase 2 (*Malus × domestica*)	XM_008374895.3	2e^−84^	other (purine synthesis)
M70	Transcription factor MYC2-like (*Malus × domestica*)	XM_008343741.3	3e^−133^	gene expression regulation
M71	S-formylglutathione hydrolase (*Nicotiana* × *attenuata*)	XM_019391580.1	1e^−08^	other (cell detoxification)
M75	Alpha/beta hydrolase (*Malus × domestica*)	WP_105934285.1	1e^−33^	defense and host-pathogen interaction
M77	Histone H3.3 (*Malus × domestica*)	XM_008345103.3	1e^−48^	gene expression regulation
M79	Far upstream element-binding protein 1-like isoform X2 (*Malus × domestica*)	XP_029106921.1	6e^−32^	gene expression regulation
M9	LHC-I protein complex (*Nicotiana* × *tabacum*)	X64198.1	4e^−112^	other (photosynthesis)

## Data Availability

The data presented in this study are available on request from the corresponding author.

## Supplementary Materials

The following are available online at https://www.mdpi.com/1422-0067/22/2/527/s1.
