# Supplementary material for: Apple Autotetraploids with Enhanced Resistance to Apple Scab (Venturia inaequalis) Due to Genome Duplication-Phenotypic and Genetic Evaluation
Source: Int J Mol Sci. 2021 Jan 7;22(2):527. doi: 10.3390/ijms22020527 (PMC7825683; doi:10.3390/ijms22020527)
Supplement: Supplementary file 1 [file ijms-22-00527-s001.pdf]

Table S1. Primers used in apple 'Free Redstar' plants analyses

No.	Primers	Primers' sequence (F/R 5'-3')
**AFLP* / cDNA-AFLP**		
1	Pst-AT/Mse-AT*	GACTGCGTACATGCAGAT / GATGAGTCCTGAGTAAAT
2	Pst-AT/Mse-TC*	GACTGCGTACATGCAGAT / GATGAGTCCTGAGTAATC
3	Pst-AA/Mse-AC*	GACTGCGTACATGCAGAA / GATGAGTCCTGAGTAAAC
4	Pst-AC/Mse-AC*	GACTGCGTACATGCAGAC / GATGAGTCCTGAGTAAAC
5	Pst-AC/Mse-AA*	GACTGCGTACATGCAGAC / GATGAGTCCTGAGTAAAA
6	Pst-TC/ Mse-TC	GACTGCGTACATGCAGTC / GATGAGTCCTGAGTAATC
7	Pst-TC/ Mse-AT	GACTGCGTACATGCAGTC / GATGAGTCCTGAGTAAAT
8	Pst-AA/ Mse-AA	GACTGCGTACATGCAGAA / GATGAGTCCTGAGTAAAA
9	Pst-GG/ Mse-GG	GACTGCGTACATGCAGGG / GATGAGTCCTGAGTAAGG
10	Pst-GG/ Mse-GA	GACTGCGTACATGCAGGG / GATGAGTCCTGAGTAAGA
**MSAP**		
11	E-AG/MH-ATG	GACTGCGTACCAATTCAAG / GATGAGTCCTGAGTCGGATG
12	E-AG/MH-CTC	GACTGCGTACCAATTCAAG / GATGAGTCCTGAGTCGGCTC
13	E-AG/MH-CAT	GACTGCGTACCAATTCAAG / GATGAGTCCTGAGTCGGCAT
14	E-AG/MH-GT	GACTGCGTACCAATTCAAG / GATGAGTCCTGAGTCGGGT
15	E-AT/MH-ATG	GACTGCGTACCAATTCAAT / GATGAGTCCTGAGTCGGATG
16	E-AT/MH-CA	GACTGCGTACCAATTCAAT / GATGAGTCCTGAGTCGGCA
**PCR for *Rvi* genes**		
17	Vm	CCTTGACGCAGCTT / CCTTGACGCATCTACG
18	ACS7	GTGCCAATGTAATCAGAGTGACGTG / ATGTAGGTGGTGATGTATCTGGATT
19	ACS9	ACATGGAAGATGAAGGAGAAGGAG / GATAAATTGAGTGACTGCAAAGCG
20	Rvi7	AAACTGAAGCCATGAGGGC / TCAATTCACATGAGGCTG
21	OBP12	CCACAGCAGTCATTGGGA/ CCACAGCAGTGCATAAAC
22	OPL19	ACCTGCACTACAATCTTCACTAATC / GACTCGTTTCCACTGAGGATATTTG
23	K08	GAACACTGGGCAAAGGAAAC / TAAAAGCCACGTTCTCTCGC
24	T06	CGTTCAACTCATAAGTGGTCC / AAGGGCAGAATGATAAAAGCC
25	Z13	CCCTAGCATGCCATAAAACC / CCCAGTGGAATATTTCGAGG
26	HB09	GCTCAAAATACTGAAGCCTTGC / GGGGAAGCAGGATGGTTACTCT
27	AD13	GGTTCCTCTGTAAAGCTAG / GGTTCCTCTGCCCAACAA
28	CH-Vf1	ATCACCACCAGCAGCAAAG / CATACAAATCAAAGCACAACCC
**real-time PCR**		
29	MdCKB4	ACATCGAGAGCTTTGGCTAAC / CTCCATCAGAACCACTGACATC
30	PR1	CGGTGAAAACCTTGCCATGA / ACTGCTGCACCTCACTTTTG
31	PR2	ACATCGCTGTTGGAAACGAAGTGC / GCCTTGCAGATTGGCTGCTACAAT
32	Rvi6	AACTAGTTTTTGGGGTATGGTTGC / CGTTACCAAGATTGCATTCTCTGT
33	WRKY29	CGTCGAAACTCTCTTGCTGG / GCTTTCCACAAACTCACGGT
34	CDPK	TCAGCGGTGTACCTCCATTT / CTACTCCGCCTACTCTGAC
35	MPK4	CAATGACACGAGAGCCCTTG / GGGTACTGTGGAAGCTGTCT

Table S2. AFLP analysis of genetic changes in tetraploids of apple 'Free Redstar' with respect to diploid counterpart

AFLP primer pair	Tetraploid clone	Number of AFLP markers		AFLP product length (bp)	Tetraploid clone	Number of AFLP markers		AFLP product length (bp)
		Monomorphic	Polymorphic			Monomorphic	Polymorphic	
Pst-AT/Mse-AT	4x-1	43	0	700-120	4x-3	46	2	700-120
Pst-AT/Mse-TC		42	1	650-200		43	0	650-200
Pst-AA/Mse-AC		26	0	700-150		24	0	700-150
Pst-AC/Mse-AC		39	3	700-200		44	0	700-200
Pst-AC/Mse-AA		39	3	600-200		33	0	600-200
	**Total**	**182**	**7**		**Total**	**190**	**2**	
Pst-AT/Mse-AT	4x-2	46	3	700-120	4x-5	43	2	700-120
Pst-AT/Mse-TC		43	0	650-200		42	0	650-200
Pst-AA/Mse-AC		24	0	700-150		26	0	700-150
Pst-AC/Mse-AC		44	0	700-200		39	3	700-200
Pst-AC/Mse-AA		33	0	600-200		39	3	600-200
	**Total**	**187**	**3**		**Total**	**181**	**8**	

Table S3. Analysis of presence of *Rvi* genes in tetraploid genotypes of apple 'Free Redstar' compared to diploid (2x) counterpart and 'Idared' reference sensitive to apple scab

The tested gene, primers and PCR products (bp)		The tested genotypes				
		**4x-1**	**4x-2**	**4x-3**	**FR2x**	**Ida2x**
Rvi5 (Vm)	Vm	690 (R)	690 (R)	690 (R)	690 (R)	–
Rvi6 (Vf)	ACS7	320 (R)	320 (R)	320 (R)	–	–
	ACS9	490 (R)	490 (R)	490 (R)	490 (R)	–
Rvi7 (Vfh)	Rvi7	250	250	250	250	250
Rvi8 (Vh8)	OBP12	200, 500, 630, 800 (R)	200, 500, 630, 800 (R)	200, 500, 630, 800 (R)	200, 500, 630, 800 (R)	630
	OPL19	500 (R), 1000	500 (R), 1000	500 (R), 1000	500 (R), 1000	1100
Rvi11 (Vbj)	K08	740 (R), 900	740 (R), 900	740 (R), 900	740 (R), 900	900
	T06	790	790	790	790	–
	Z13	770 (R)	770 (R)	770 (R)	770 (R)	–
Rvi14 (Vdr1)	HB09	210 (R), 250	210 (R), 250	210 (R), 250	210 (R), 250	200, 250, 400
Rvi15 (Vr)	AD13	1100	1100	1100	1100	–
Rvi17 (Va1)	CH-Vf1	160 (R), 300, 400	160 (R), 300, 400	160 (R), 300, 400	160 (R), 300, 400, 500	100, 500

R sequence associated with resistance to apple scab
– no PCR products
